# Effective removal of Cr(vi) from aqueous solution by biochar supported manganese sulfide†

**DOI:** 10.1039/c9ra06028f

**Published:** 2019-10-02

**Authors:** Shiqiu Zhang, Haiqing Zhang, Fang Liu, Fan Yang, Shengnan Zhou, Kui Zheng, Chunli Chu, Le Liu, Meiting Ju

**Affiliations:** College of Environmental Science and Engineering, Nankai University 38# Tongyan Road, Jinnan District 300350 Tianjin PR China nkujumeiting@sohu.com tjliule@126.com +86 13672031215 +86 13820988813; Huaxin College of Hebei Geo University Shijiazhuang 050031 Hebei PR China; Key Laboratory of Cardiovascular Remodeling and Function Research, Qilu Hospital, Shandong University Jinan 250014 Shandong PR China; Analytical and Testing Center, Southwest University of Science and Technology Mianyang 621010 Sichuan PR China

## Abstract

In order to remove hexavalent chromium (Cr(vi)) efficiently and simplify the adsorbent preparation process, we employed a single step method to prepare a new biochar supported manganese sulfide material. The nanoscale MnS particles were highly soldered on the biochar support surface, and this adsorbent displayed the effective removal of Cr(vi) (98.15 mg L^−1^) *via* synergistic effect between adsorption and reduction/precipitation under weak acid conditions (pH = 5.0–6.0). The adsorption kinetic data were described well by the pseudo second-order kinetic model, suggesting that the reaction process was a chemisorption process. The adsorption isotherm data were described well by the Redlich–Peterson model, further suggesting that this reaction was a hybrid chemical reaction-sorption process. In addition, the Dubinin–Radushkevich isotherm model with 8.28, 8.57, and 12.91 kJ mol^−1^ adsorption energy also suggests that it was a chemisorption process. The simple and eco-friendly preparation process, low-cost, and the high removal efficiency could make it a promising material for the purification of Cr(vi)-contaminated wastewater.

## Abstract 2

In order to remove hexavalent chromium (Cr(vi)) efficiently and simplify the adsorbent preparation process, we employed a single step method to prepare a new biochar supported manganese sulfide material.

## Graphical abstract

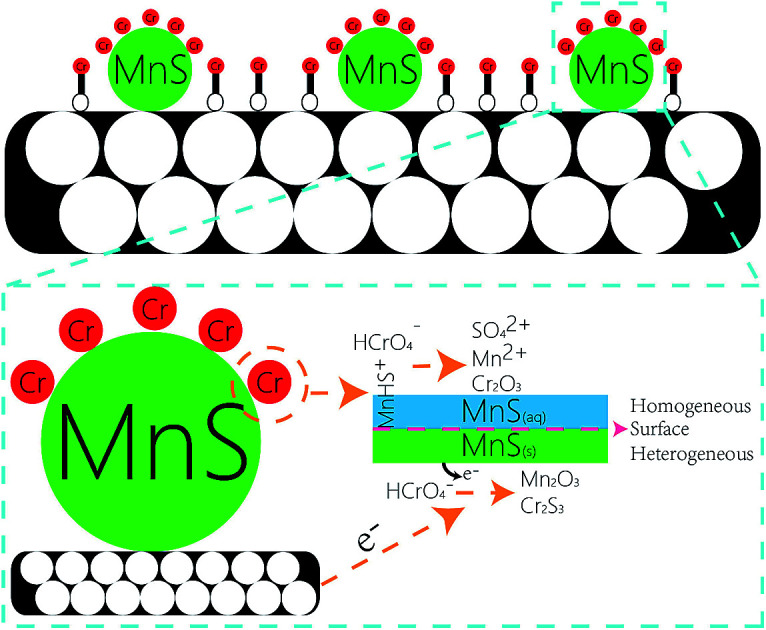

## 1. Introduction

It is well known that there are two forms of chromium (Cr) element: trivalent chromium (Cr(iii)) and hexavalent chromium (Cr(vi)).^1^ Cr(iii) is less toxic and mostly in the form of precipitates, and a trace of Cr(iii) is essential for animals and plants, as it is responsible for the control of lipid metabolism and glucose conversion.^2^ While, Cr(vi) is mostly in the form of soluble and mobile oxyanions with hyper-toxicity (about 300 times toxic than Cr(iii)), due to the excellent mobility and bioavailability.^3^ As one of most toxic heavy metal elements in water, Cr(vi) could stay in the organism for a long period, resulting in a high risk of cancers, due to carcinogenicity, persistence, and bioaccumulation.^4,5^ In general, Cr-containing sewage is often derived from industry discharge, *e.g.* electroplating industry, textile industry, curriery, and metallurgy.^6^ The United States Environmental Protection Agency (USEPA) has identified Cr(vi) as a top priority hazardous contaminant, and the mandatory discharge limits of Cr(vi) are 500 μg L^−1^ for waste water and 50 μg L^−1^ for drinking water.^7,8^ However, the Cr(vi) concentrations in waste water usually range from 30 mg L^−1^ to 200 mg L^−1^.^9^

Many technologies have been investigated to remove or reduce Cr(vi) from waste water in the published researches, *e.g.* physical methods (membrane separation, ultrafiltration, reverse osmosis, and adsorption), chemical methods (chemical precipitation, oxidation/reduction, ion exchange, adsorption) and biological methods (microorganism).^10^ Among these technologies, adsorption method has been agreed to be the frontline for Cr(vi) removal from waste water due to its outstanding characteristics, *e.g.* handleability, high selectivity, economic efficiency, and environmentally friendly.^11^ Considering the development of this method, the low cost and high efficient adsorbent is the primary concern.^12,13^ Based on the researches, biochar has attracted widely attentions as the excellent Cr(vi) adsorption material due to its high surface area and stability, abundant aperture structure and surface functional groups,^14,15^ and corn straw is one of the common biomass waste to prepare the biochar with excellent physicochemical properties,^16^*e.g.* high O/C ratio, cation-exchange capacity, abundant surface functional groups, and large specific surface area. According to the published literatures,^7,17^ there are four mechanisms for the removal of Cr(vi) from waste water by biochar support materials. First one is the electrostatic adsorption. During the process, Cr(vi) cation could be adsorbed onto the charged biochar surface without reduction reaction. While, this form of Cr(vi) removal is relatively ineffective. Another one is the cationic adsorption. For this pathway, all of Cr(vi) could be reduced to Cr(iii) and then Cr(iii) would be adsorbed onto the biochar surface. The third pathway is complete reduction, which Cr(vi) could be adsorbed onto the biochar material surface and then completely reduced to Cr(iii). The last one is the combination of adsorption and reduction. During the process, a part of Cr(vi) would be adsorbed onto biochar materials surface and the others would be reduced to Cr(iii). In order to meet strict environmental regulations, Cr(vi) should be effectively adsorbed onto the biochar materials and then reduced to Cr(iii) as the combination of adsorption and reduction reveals the excellent removal efficiency.^18^ For the reduction of Cr(vi) in waste water, the typical reducing agents are iron or iron(ii) ions and the reaction is usually executed under strong acidic conditions (pH = 1 or 2).^20,21^ Moreover, the reported material preparation methods are usually two-step pathway (including biochar preparation and biochar modification), which requires large amounts of chemical reagents, high energy consumption, and complex preparation process, leading to the high preparation cost and secondary environmental pollution.^8,18,22^ Especially in the published word by Lyu *et al.*, the dissolution of FeS nanoparticles was near 80% after Cr(vi) adsorption.^18^ Moreover, during the CMC-FeS@biochar preparation process, the water should be purged with purified N_2_, and the whole devices needed the N_2_ protection, resulting a complex procedure and high cost. Wadhawan^19^ proved that Cr(vi) could be reduced by MnS effectively and the preparation process was very simple *via* hydrothermal method. However, to our knowledge, there were no published works reported about the preparation of biochar-supported MnS nanoparticles (MnS@biochar), and there was also no detailed investigation into the effectiveness of Cr(vi) removal by MnS@biochar.

In this work, the overall goal was to prepare a new biochar support MnS (MnS@biochar) material *via* one-pot method to adsorb and reduce Cr(vi) in aqueous solution. In the experimental design, MnS loaded dosage, MnS@biochar dosage, solution pH, retention time, and initial Cr(vi) concentrations were executed to test the Cr(vi) removal capacity of MnS@biochar. The characterizations and mechanism were analyzed by transmission electron microscope (TEM), scanning electron microscope (SEM), X-ray diffraction (XRD) patterns, Fourier transform infrared spectroscopy (FT-IR), ζ-potential analysis, and X-ray photoelectron spectroscopy (XPS).

## 2. Materials and methods

### 2.1 Reagents

Manganese acetate (C_4_H_14_MnO_8_, AR: 99%), and ethylenediamine (EDA, C_2_H_8_N_2_, AR: 99%) were purchased from Tianjin Kemiou Chemical Reagent Co., Ltd. Thioacetamide (CH_3_CSNH_2_, AR: 99%) was purchased from Sinopharm Chemical Reagent. Potassium dichromate (K_2_Cr_2_O_7_, AR: 99%) was purchased from Aladdin Chemistry Co. Ltd. The deionized water with a resistivity of 18.25 MΩ cm was used for all the experiments.

### 2.2 MnS@biochar preparation

The biomass used in the experiment was corn straw, which obtained from an agricultural area in Ninghe, Tianjin. The raw material was washed with deionized water and dried in air. Subsequently, the sample was grind passed through a 0.5 mm mesh size sieve. For MnS@biochar preparation, 0.4684 g of manganese acetate, the corresponding amounts of thioacetamide, 5 mL of EDA, and 60 mL of deionized water were added into a 200 mL of conical flask, and then the mixture was vigorously stirred for 120 min. Then, 0.5, 1.0, 2.5, 5.0, and 10.0 g of corn straw were added into the mixture, respectively, and the stirring was executed for another 30 min. Next, the mixture was transferred into 100 mL of autoclave, and heated at 245 °C for 18 h. Lastly, the product was filtrated and washed by deionized water and methanol for several times, and dried under vacuum at 373 K for 12 h. The samples were named as MnS@biochar-1, MnS@biochar-2, MnS@biochar-3, MnS@biochar-4, and MnS@biochar-5, respectively.

### 2.3 Batch experiments

All of the batch experiments were executed in 100 mL of conical flask in an air thermostatic shaker (HNYC-2102C, Honour Instrument, Tianjin). In detail, a series of parameters were carried out, including MnS loaded dosage, MnS@biochar dosage (0.5–3.0 g L^−1^), solution pH value (2–10), retention time (0–1440 min), and initial Cr(vi) concentrations (50–300 mg L^−1^). For the MnS loaded dosage, 0.1 g of MnS@biochar-(1–5) sample and 50.00 mL of Cr(vi) aqueous solution (*C*_Cr(VI)_ = 150.00 mg L^−1^) were placed in 100 mL of conical flask for 1440 min, and the pH value was kept at 5.0–6.0 (25 °C) using HCl (0.1 mol L^−1^) or NaOH (1 mol L^−1^). For the effect of MnS@biochar dosage, the various amounts of the MnS@biochar and 50.00 mL of Cr(vi) aqueous solution (*C*_Cr(VI)_ = 150.00 mg L^−1^) were placed in 100 mL of conical flask for 1440 min, and the pH value was kept at 5.0–6.0 (25 °C) using HCl (0.1 mol L^−1^) or NaOH (1 mol L^−1^). For the effect of solution pH value, the mixture was adjusted to the range of 2–10 (25 °C) using HCl (0.1 mol L^−1^) or NaOH (1 mol L^−1^). To examine the effect of retention time, 75.00 mg of MnS@biochar and 50.00 mL of Cr(vi) aqueous solution (*C*_Cr(VI)_ = 150.00 mg L^−1^) were placed in 100 mL of conical flask for predetermined time, and the pH was kept at 5.0–6.0 (25 °C) using HCl (0.1 mol L^−1^) or NaOH (1 mol L^−1^). To determine the adsorption isotherms, different initial concentrations of Cr(vi) solution (50–300 mg L^−1^, 50.00 mg L^−1^ interval) were agitated until equilibrium was achieved with the same adsorbent dosage in 100 mL of conical flask with solution pH value of 5.0–6.0 (25 °C) using HCl (0.1 mol L^−1^) or NaOH (1 mol L^−1^). Moreover, the adsorption temperature (15, 25, and 35 °C) was also executed. After the adsorption process, the suspension was filtered through a 0.45 μm cellulose acetate membrane filter paper, and the Cr(vi) concentration in the filtrate was analyzed using an Elan drc-e ICP-MS (PerkinElmer, USA). The average data were reported after three times repeated experiments, and the blank sample without adsorbent was also carried out to verify the insignificance of Cr(vi) degradation and adsorption to the conical flask.

### 2.4 Data analysis

#### 2.4.1 Equilibrium adsorption amount

The equation of equilibrium adsorption amount was as follows:^23^
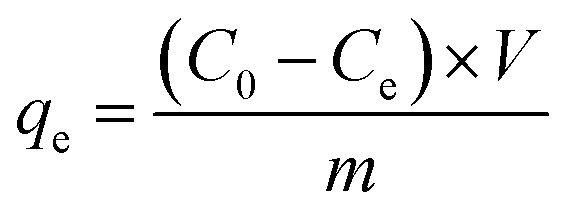
where, *q*_e_ was Cr(vi) equilibrium adsorption amounts (mg) of per gram MnS@biochar (mg g^−1^), *C*_0_ was the initial concentration of Cr(vi) (mg L^−1^), *C*_e_ was the concentration of Cr(vi) at equilibrium stage (mg L^−1^), *V* was the volume of Cr(vi) solution (L), *m* was the weight of MnS@biochar (g).

#### 2.4.2 Adsorption kinetics analysis

The equation of pseudo-first-order (PFO) kinetic model was as follows:^23^
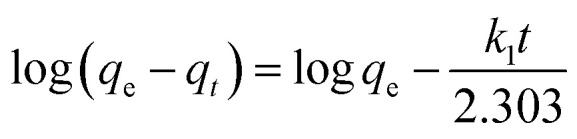


The equation of pseudo-second-order (PSO) kinetic model was as follows:^22^
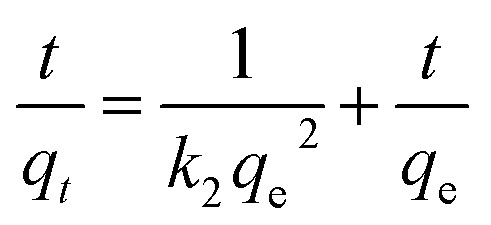
where, *k*_1_ was equilibrium rate constant of PFO kinetic model (min^−1^), *k*_2_ was the constant of PSO kinetic model (g (mg^−1^ min^−1^)), *q*_e_ was the Cr(vi) adsorption amount (mg g^−1^) at equilibrium time, *q*_*t*_ was the Cr(vi) adsorption amount (mg g^−1^) at any time *t* (min), *k*_2_ was determined by plotting *t*/*q*_*t*_*versus t*.

#### 2.4.3 Adsorption thermodynamics analysis

The equation of Langmuir adsorption isotherm model was as follows:^24^
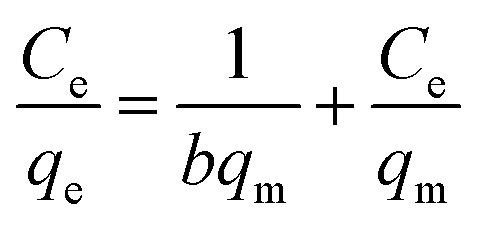


The equation of Freundlich adsorption isotherm model was as follows:^24^
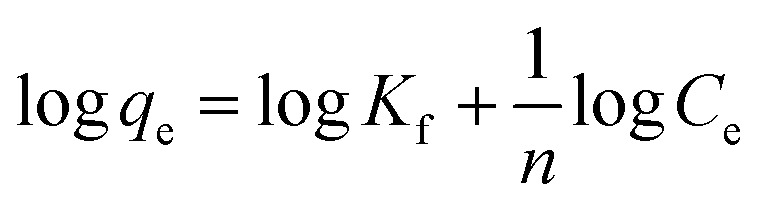


The equation of Redlich–Peterson (RP) adsorption isotherm model was as follows:^4^
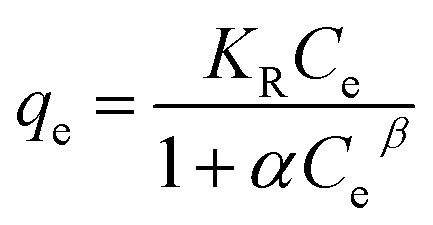


The equation of Dubinin–Radushkuvich (DR) adsorption isotherm model was as follows:^25^ln *q*_e_ = ln *q*_m_ − *K*_D_*ε*^2^where, *C*_e_ was the concentration of Cr(vi) at equilibrium stage (mg L^−1^), *q*_e_ was the adsorption amount of Cr(vi) on MnS@biochar at equilibrium stage (mg L^−1^), *q*_m_ was the monolayer adsorption capacity of MnS@biochar (mg L^−1^), *b* was the Langmuir constant related to the affinity of the bending sites and energy of adsorption (L mg^−1^), *K*_R_ and *α* were the constants of DP isotherm model, *β* was the exponent ranged from 0 to 1, *K*_D_ was the constant of DR isotherm model [mol^2^ kJ^−2^], *ε* was the Polanyi potential energy (kJ mol^−1^). *ε* = 2.48 ln(1 + 1/*C*_e_), *q*_m_ and *b* could be obtained from the slope and intercepts of the linear plots of *C*_e_/*q*_e_*versus C*_e_, *K*_f_ was the Freundlich constant indicted the adsorption capacity and *n* was another Freundlich constant indicated the adsorption intensity.

### 2.5 Characterization

The morphology analysis of MnS@biochar was characterized by scanning electron microscopy (SEM) (UItra55, Carl zeiss irts Corp., Germany). The size distributions and morphology of MnS particles on biochar were determined by transmission electron microscopy (TEM, Libra200, Carl zeiss irts Corp., Germany) at accelerating voltage of 120 kV. The structural characterizations of biochar, MnS, fresh MnS@biochar (F–MnS@biochar) and MnS@biochar after adsorption (R–MnS@biochar) were tested by X-ray diffraction (XRD) (PANalytical, X'Pert PRO) with a Cu-Kα (*λ* = 0.15418 nm) radiation source. The contents of C element, H element, N element, and S element in biochar and F–MnS@biochar were measured by Euro EA3000 Elemental Analyzer (Leeman, USA), and the average data were reported after twice repeated experiments. For specific surface areas (SSA) analysis, the biochar, MnS, and F–MnS@biochar samples were determined by the ChemiSorb 2720 Analyzer (Micromeritics Instrument, USA). The samples were outgassed at 105 °C for 16 h and then executed the N_2_ adsorption at 77 K. The Fourier transform infrared spectroscopy (FT-IR) were collected by the Spectrum One FT-IR spectrometer ranged from 4000 cm^−1^ to 400 cm^−1^ with average 32 scans at resolution of 2 cm^−1^ (PerkinElmer, Waltham, MA, USA). The zeta-potential of biochar, MnS, and F–MnS@biochar samples were performed at 25 °C by Zetasizer Nano (Zs90, Malvern Instruments, UK). The X-ray photoelectron spectroscopy (XPS) analysis was recorded by the XPS system (Kratos AXIS Ultra) at 150 W equipped with a monochromatic Al X-ray source. The analysis of the XPS spectra was executed by XPSPEAK software (version 4.1).^26^

## 3. Results and discussions

### 3.1 Adsorption results

#### 3.1.1 Effects of adsorption parameter

**Fig. 1:**
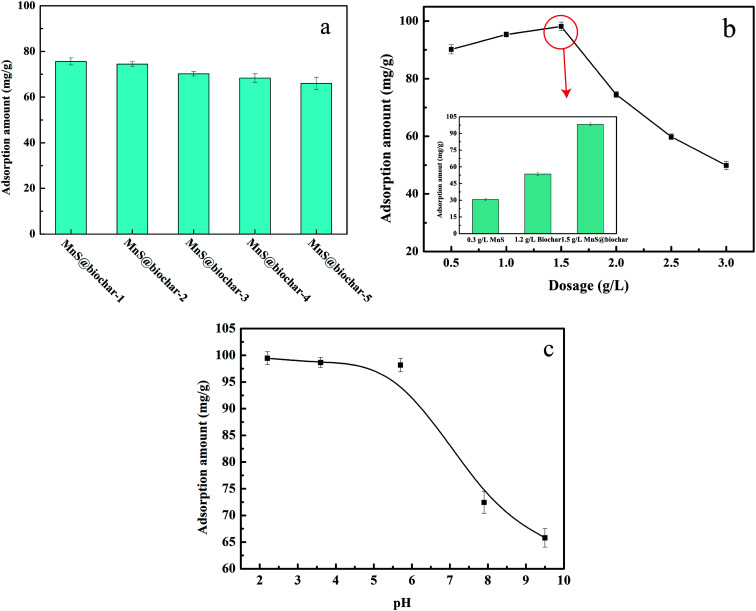
Removal capacities of MnS@biochar ((a) effect of MnS loaded dosage; (b) effect of MnS@biochar dosage; (c) effect of solution pH value).

The equilibrium Cr(vi) adsorption capacities of MnS@biochar sample with different MnS loaded dosages, different dosages and solution pH values were shown in Fig. 1. As shown in Fig. 1a, it released that the equilibrium Cr(vi) adsorption capacity decreased with MnS loaded dosages decreased, and the value of MnS@biochar-1 was closed to that of MnS@biochar-2. Considering the equilibrium Cr(vi) adsorption amount and MnS@biochar yield, the MnS@biochar-2 sample was selected as the final experimental adsorbent, denoted as MnS@biochar. As shown in Fig. 1b, it indicated that the increasing MnS@biochar dosage from 0.5 g L^−1^ to 1.5 g L^−1^ caused an increase in Cr(vi) removal amounts (from 90.18 mg g^−1^ to 98.15 mg g^−1^). While, when the adsorbent dosage increased from 1.5 g L^−1^ to 3.0 g L^−1^, the equilibrium Cr(vi) removal capacities of MnS@biochar decreased to 49.84 mg g^−1^, which could meet the maximum contaminant level of 0.5 mg L^−1^ for Cr(vi) in waste water. Compared to 1.5 g L^−1^ of MnS@biochar, Cr(vi) removal amounts of MnS (0.3 g L^−1^) and biochar (1.2 g L^−1^) were 30.61 mg g^−1^ and 53.53 mg g^−1^, suggesting that there was a synergistic effect between MnS particles and biochar support. As shown in Fig. 1c, the solution pH value also played an important key role in the Cr(vi) removal from aqueous solution, and the optimal solution pH was the acidic environment. On the other hand, based on the CrO_4_^2−^ hydrolysis (Fig. S1†), the reduced repulsive force between Cr(vi) and negative charged MnS@biochar would be also contribution to the increased Cr(vi) removal amount during the acidic adsorption solution.^18^

#### 3.1.2 Adsorption kinetics

**Fig. 2:**
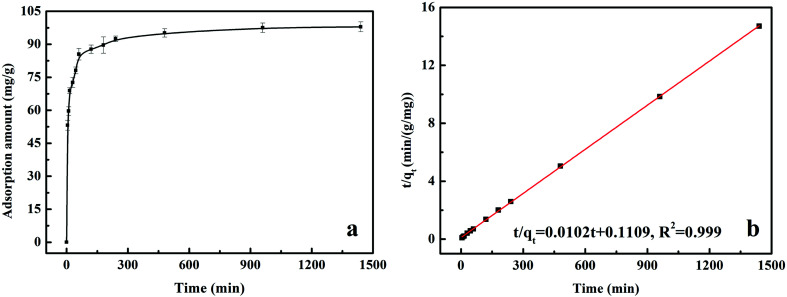
The relationship between adsorption amount and retention time (a); pseudo second-order kinetic model fitting line (b).

Fig. 2a showed the relationship between Cr(vi) removal amount and adsorption retention time. From the results, it suggested that the adsorption rate was rapid within 60 min, and then it slowed down until the equilibrium state was reached at about 480 min. In addition, as shown in Fig. 2b and S2,† the adsorption data could be matched very well by pseudo-second-order (PSO) kinetic model, suggesting that Cr(vi) adsorption on MnS@biochar was a chemical process occurring on MnS@biochar surface.^16^ The fitting parameters of PFO kinetic model and PSO kinetic model were listed in Table S1.† The correlation coefficient (*R*^2^) of PSO kinetic model was 0.999, which was higher than 0.925 for PFO kinetic model. The *q*_e_ value calculated by PSO kinetic model was closed to the experimental result. Moreover, the normalized standard deviation (NSD) and average relative error (ARE) values of PSO kinetic model were lower than these of PFO kinetic model, further suggesting that the adsorption data could be matched very well by PSO kinetic model. Compared to the published works (Table S2†), the adsorption capacity of MnS@biochar was also at a high level.

#### 3.1.3 Adsorption isotherm

**Fig. 3:**
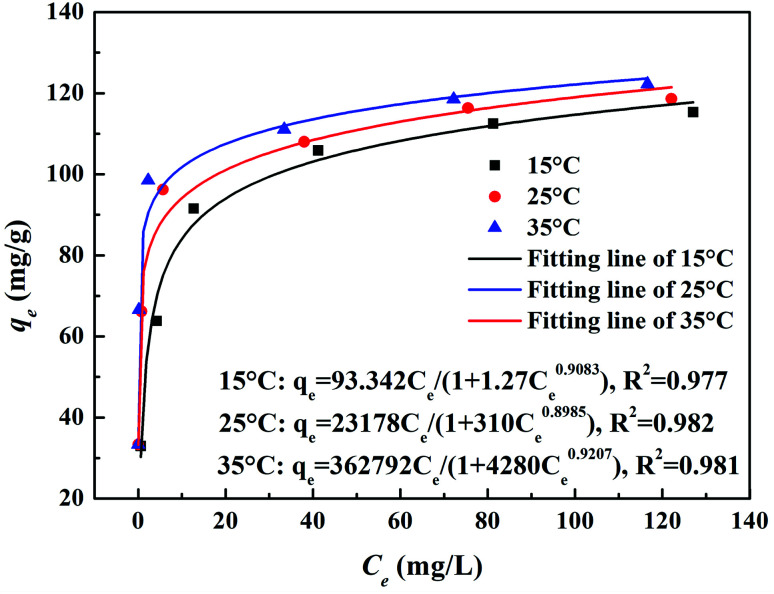
The relationship between removal amount and initial Cr(vi) concentration and the fitting results of Redlich–Peterson model.

The adsorption isotherm was another important factor in describing the interactive behavior between Cr(vi) and MnS@biochar. Fig. 3 showed the Cr(vi) removal amounts of MnS@biochar at different initial Cr(vi) concentration and adsorption temperature, and the models of Langmuir (Fig. S3a†), Freundlich (Fig. S3b†), Redlich–Peterson (Fig. 3), and Dubinin–Radushkuvich (Fig. S3c†) were fitted. As shown in Fig. 3, it was evident that the Cr(vi) removal capacity increased with the initial Cr(vi) concentration increased, and the higher adsorption temperature would enhance the Cr(vi) removal capacity of MnS@biochar. The fitting parameters of these adsorption isotherm models were listed in Table S3.† The results showed that both of Langmuir model and RP model had the higher *R*^2^ (above 0.980) at different adsorption temperature, and RP model had the lowest values of MPSD and HYBRID, suggesting that Cr(vi) removal by MnS@biochar was a combination process of chemical reaction and adsorption.^23^ The *β* value of RP model was closed to 1.0, suggesting that the Cr(vi) removal by MnS@biochar process was closed to the ideal Langmuir condition. The bonding energy of Cr(vi) adsorption onto MnS@biochar was estimated by DR model (*R*^2^ > 0.88), and the values at different adsorption temperature were about 8.28, 8.57, and 12.91 kJ mol^−1^. Based on the literature,^27^ the bonding energies for chemosorption usually ranges from 8.0 kJ mol^−1^ to 16.0 kJ mol^−1^. Hence, the conclusion could further prove the chemisorption between Cr(vi) and MnS@biochar.

### 3.2 Characterizations

#### 3.2.1 Morphology analysis

**Fig. 4:**
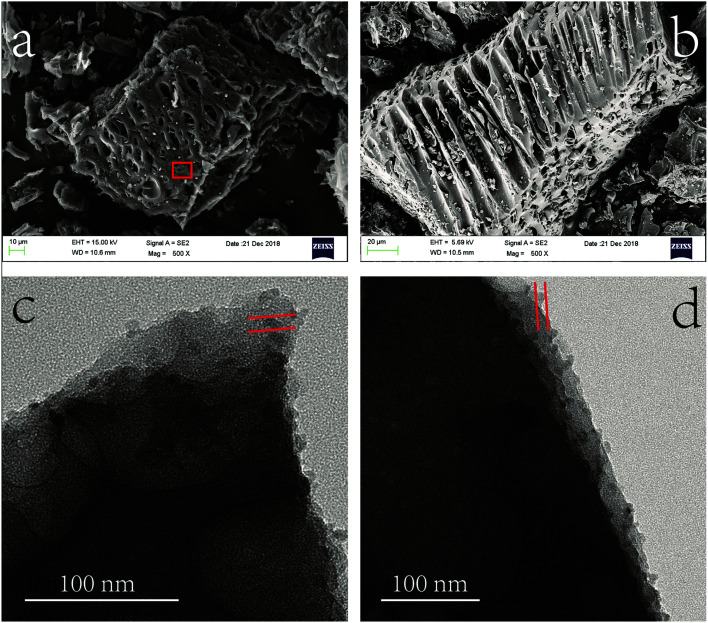
The morphology analysis of F–MnS@biochar ((a) and (b) SEM; (c) and (d) TEM).

The morphology characterizations of fresh MnS@biochar (F–MnS@biochar) *via* SEM analysis and TEM analysis were shown in Fig. 4. From Fig. 4a and b, it could be observed that biochar support presented the different morphologies of multihole structure. Moreover, the EDS analysis (Fig. S4†) could prove that Mn element and S element were distributed on biochar surface. In order to observe the morphology of MnS particle on the biochar support, TEM analysis was employed. From Fig. 4c and d, it showed that there was no specific morphology for MnS particle and it could be observed as the nearly spherical structure with nanoscale particles size (20–50 nm). Hence, both of multihole structure of biochar support and nanoscale MnS particle would be contributed to the high Cr(vi) removal capacity of MnS@biochar.

#### 3.2.2 Characterization analysis

The elements analysis results of biochar, MnS, and F–MnS@biochar were listed in Table 1. For biochar sample, the contents of C element, H element, N element, and S element were 62.22, 3.96, 0.81, and 0.33 wt%, respectively. MnS was composed of S (33.42 wt%), which was close to the theoretical value. F–MnS@biochar were composed of C element (51.52 wt%), H element (3.48 wt%), N element (0.68 wt%), and S element (3.75 wt%). Compared to biochar sample, F–MnS@biochar had the higher ratios of O/C, and the high O-containing functional groups would play a vital role in Cr(vi) removal. For the specific surface areas and porous structures of MnS, biochar, and F–MnS@biochar (Fig. S5† and Table 1), biochar and F–MnS@biochar presented the largest SSA and type II isotherm with the H4 type hysteresis loop, and MnS showed the type III isotherm with the H3 type hysteresis loop. The distributions of pore size showed that the mesoporous sizes of the biochar, MnS, and F–MnS@biochar were 3.1 nm, 15.8 nm, and 8.6 nm, respectively. The ζ-potentials of biochar, MnS, and F–MnS@biochar were shown in Fig. S6.† From the figure, it could be observed that the points of zero charges (PZCs) of biochar, and MnS were located at around pH 4.4 and 5.0, while the PZCs of F–MnS@biochar was less than pH 2.0. Compared to biochar and MnS, the F–MnS@biochar sample was obviously negative shift, which may be caused by the interactions between MnS particles and biochar support.

**Table 1:** Characterization analysis results of biochar, MnS, and F–MnS@biochar

Samples	C (%)	H (%)	N (%)	S (%)	O/C	SSA (m^2^ g^−1^)	Pore size (nm)	Pore volume (cm^3^ g^−1^)
Biochar	62.22	3.96	0.81	0.33	0.39	350.3	3.1	0.35
MnS	—	—	—	33.42	—	50.3	15.8	0.06
MnS@biochar	51.52	3.48	0.68	3.75	0.41	220.8	8.6	0.18

#### 3.2.3 XRD analysis

**Fig. 5:**
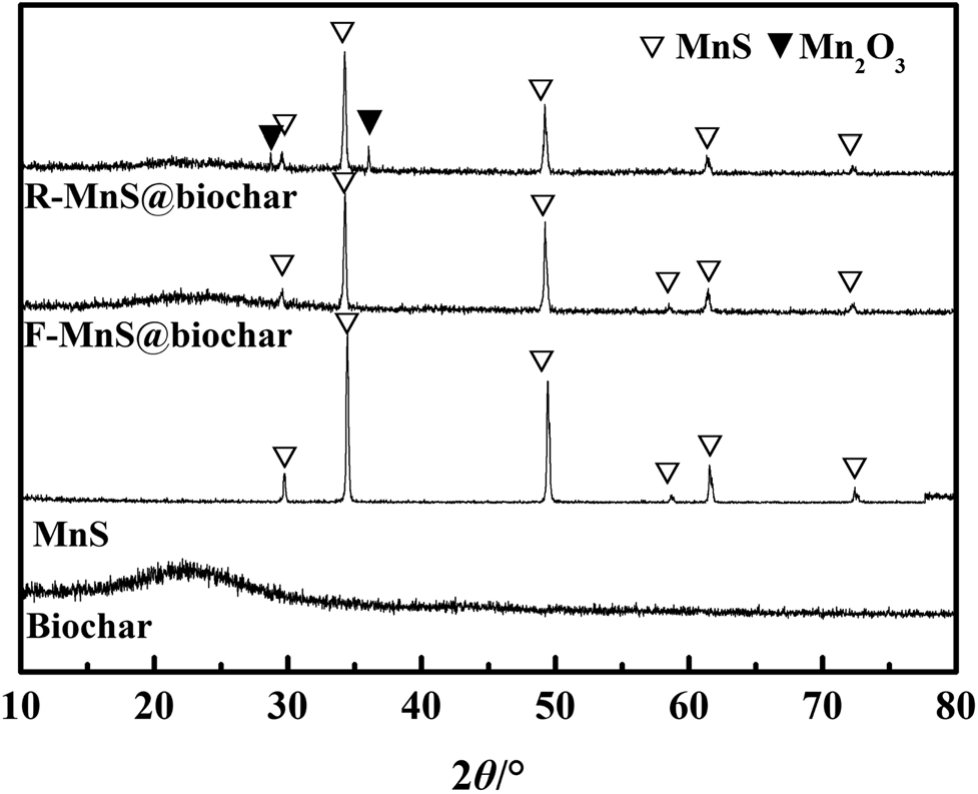
X-ray diffraction (XRD) patterns of biochar, MnS, F–MnS@biochar, and MnS@biochar after reaction (R–MnS@biochar).

The XRD patterns of biochar, MnS, F–MnS@biochar, and MnS@biochar after reaction (R–MnS@biochar) were shown in Fig. 5. From the results, we could observe the following conclusions. (1) There was a wide diffraction peak near 22.5°, which verified the presence of –OH, O=C, and C–O groups.^28^ (2) For the pattern of MnS, six characteristic peaks at 2*θ* = 29.72°, 34.46°, 49.44°, 58.72°, 61.57°, 72.43° were assigned to the indices {1,1,1}, {2,0,0}, {2,2,0}, {3,1,1}, {2,2,2}, {4,0,0} (JCPDS: no. 06-0518).^19^ (3) For F–MnS@biochar and R–MnS@biochar, the presence of the wide peak near 22.5° indicated the presence of biochar support. (4) For F–MnS@biochar, there were several diffraction peaks at 29.59°, 34.27°, 49.24°, 58.63°, 61.52°, 72.36°, which were assigned to MnS (JCPDS: no. 06-0518). (5) Compared to F–MnS@biochar, the new diffraction peaks at 28.82° and 36.06° presented the existence of hausmannite (Mn_2_O_3_, JCPDS: no. 24-0734), which was assigned to the indices {1,1,2} and {2,1,1}. The new substance would suggest that there is a chemical reaction between Cr(vi) and MnS@biochar.

#### 3.2.4 FT-IR analysis

**Fig. 6:**
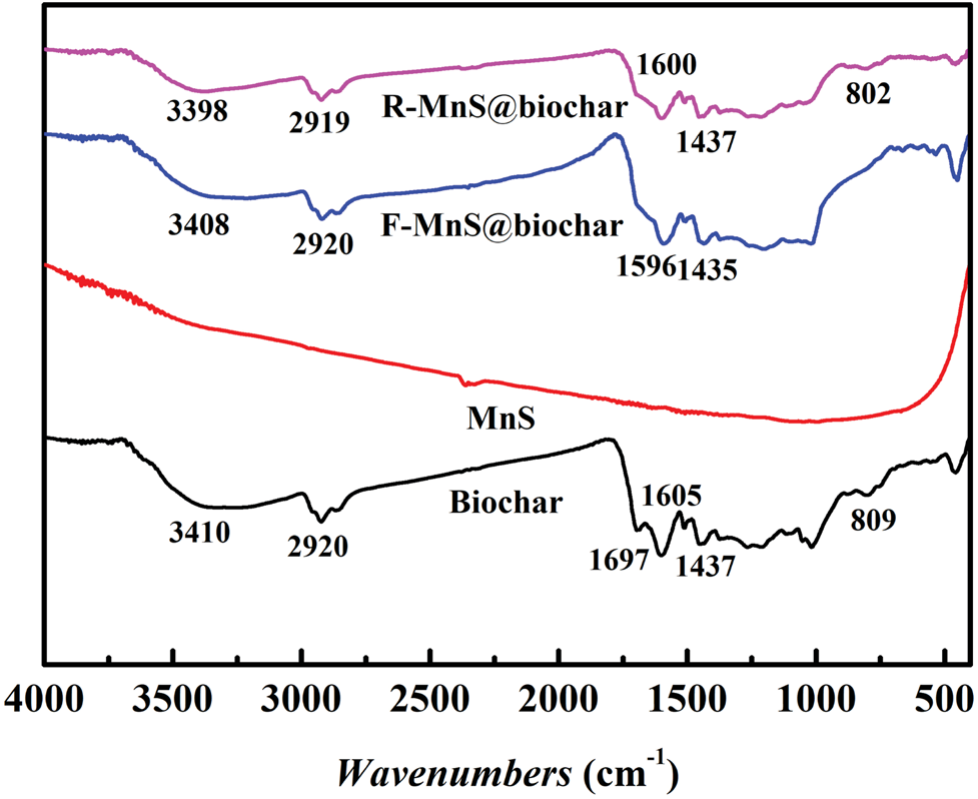
FT-IR spectra of biochar, MnS, and F–MnS@biochar, and R–MnS@biochar.

The surface functional groups of biochar, MnS, F–MnS@biochar, and R–MnS@biochar were investigated and shown in Fig. 6. From the result, it suggested that there were no obvious bands assigned to MnS. For biochar, F–MnS@biochar, and R–MnS@biochar,^23,29^ the peak near 3410 cm^−1^ was attributed to the stretching vibration of –OH; the peaks ranged from 2900 cm^−1^ to 2800 cm^−1^ were assigned to the stretching vibrations of C–H; the peak near 1437 cm^−1^ was assigned to scissoring vibrations of –CH_2_–. After adsorption, the intensity of peak near 1600 cm^−1^ assigned to the C=O stretching vibrations increased. Compared to the biochar spectra, the peaks near 1697 cm^−1^ assigned to C=C disappeared in the spectra of F–MnS@biochar and R–MnS@biochar; and the peaks near 805 cm^−1^ assigned to the Si–O stretching vibrations also disappeared in F–MnS@biochar spectra. According to the above result, it indicated that MnS particles would be soldered on biochar surface *via* the O-containing functional groups (–OH, C=C, C=O, C–O, and Si–O).

#### 3.2.5 XPS analysis

**Fig. 7:**
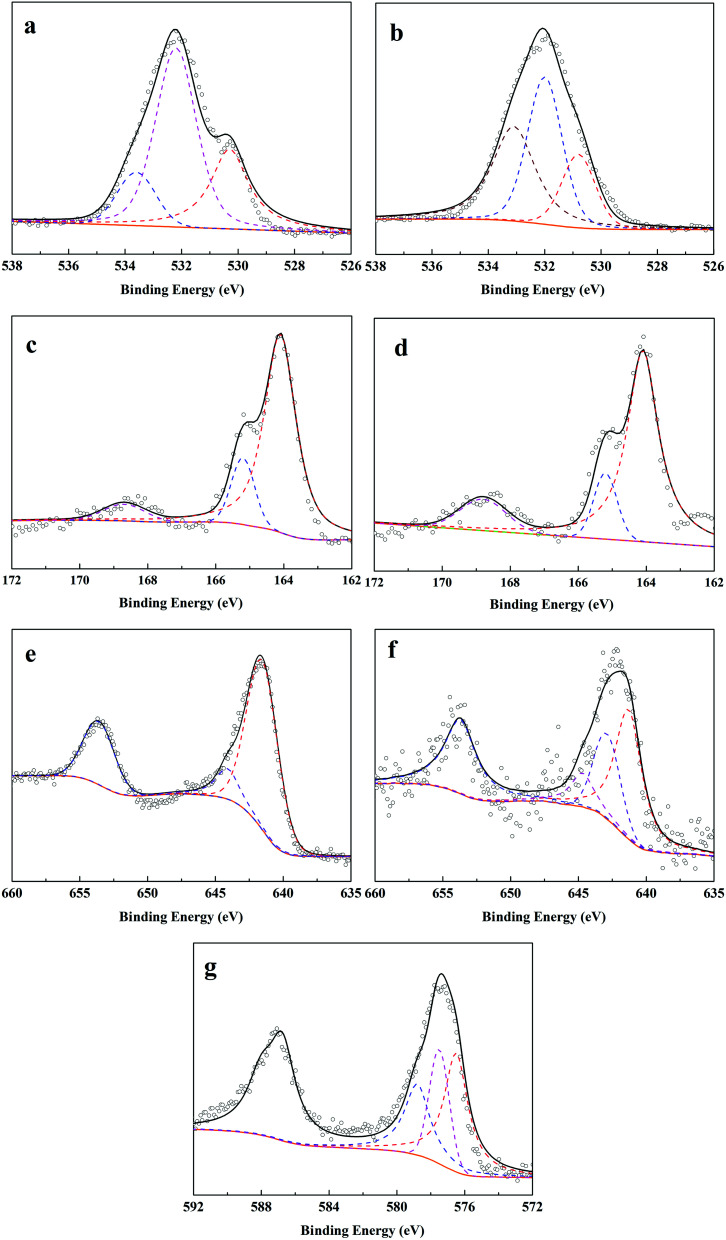
XPS spectra of F–MnS@biochar, and R–MnS@biochar ((a) O 1s spectrum on F–MnS@biochar; (b) O 1s spectrum on R–MnS@biochar; (c) S 2p spectrum on F–MnS@biochar; (d) S 2p spectrum on R–MnS@biochar; (e) Mn 2p spectrum on F–MnS@biochar; (f) Mn 2p spectrum on R–MnS@biochar; (g) Cr 2p spectrum on R–MnS@biochar).

The XPS spectra of O 1s, S 2p, Mn 2p, and Cr 2p on F–MnS@biochar, and R–MnS@biochar surface and the relevant parameters were shown in Fig. 7 and Table S4.† For the O 1s spectrum of F–MnS@biochar (Fig. 7a),^22,30,31^ the bending energies at 530.3 eV, 532.2 eV, and 533.6 eV were ascribed to C–O, –OH, and C=O, respectively. After adsorption–reduction reaction (Fig. 7b), C–O band disappeared and the percentage of –OH band decreased from 60.8% to 42.7%, while the percentage of C=O band increased from 16.9% to 35.6%. These changes suggested the complex complexation between Cr(vi) and O-contained functional groups on F–MnS@biochar surface, which was in accordance with FT-IR analysis. In addition, the new peak appeared at 530.8 eV would be ascribed to the O-contained bands in metallic oxide (*e.g.* Mn_2_O_3_ or Cr_2_O_3_).^7,19^ Considering S 2p spectra (Fig. 7c and d), the bending energies at 168.7 eV, 165.2 eV, and 164.1 eV were ascribed to S(vi)/S(iv), S(−ii) in H_2_S, and S(−ii) in MnS.^32^ However, there were no obvious changes in the S 2p_3/2_ spectra on R–MnS@biochar surface, and only the content of S element decreased from 3.7% to 2.9%. Moreover, the content of S(vi)/S(iv) increased from 7.6% to 15.3%. These changes also further proved the interaction between F–MnS@biochar and Cr(vi). For Mn 2p_3/2_ spectrum (Fig. 7e),^19^ the bending energies at 644.1 eV and 641.5 eV were attributed to Mn(ii) and Mn(iv) on F–MnS@biochar surface. While after adsorption–reduction (Fig. 7f), the new bending energy appeared at 642.9 eV should be attributed to Mn(iii), and the percentage of Mn(ii) decreased from 91.6% to 62.8%. The presence of Mn(iii) was in accordance with XRD analysis and O 1s spectra. These changes further suggested the reactions between Cr(vi) and MnS on F–MnS@biochar surface. Furthermore, for Cr(vi) 2p_3/2_ spectrum on R–MnS@biochar (Fig. 7g),^33,34^ it released that the bending energies at 578.8 eV, 577.5 eV, and 576.5 eV were attributed to Cr_2_O_3_, Cr(vi), and Cr_2_S_3_ respectively. The content of Cr element on MnS@biochar surface was about 8.7%, which was closed to the equilibrium Cr(vi) absorption amount. These data would also prove the redox reaction between Cr(vi) and F–MnS@biochar.

#### 3.2.6 Cr(vi) removal mechanism

**Fig. 8:**
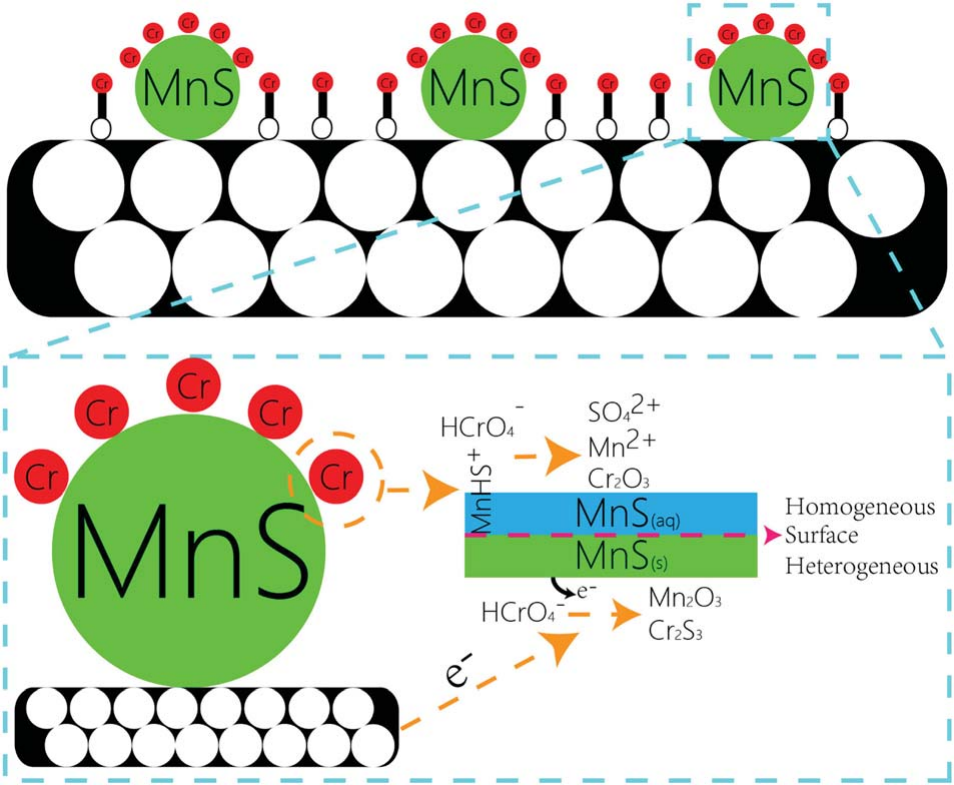
Proposed mechanism of Cr(vi) adsorption–reduction on MnS@biochar.

According to the results of XRD, FT-IR, XPS and log C-pH, we proposed a possible Cr(vi) removal mechanism by MnS@biochar as follows (Fig. 8). A portion of Cr(vi) were chemisorbed onto MnS@biochar surface through O-containing functional groups, including –OH, C=O, O=C–O, C–O, and the surface multihole structure was also contributed to the high Cr(vi) adsorption amount. The other portion of Cr(vi) were reacted with MnS nanoparticles. Generally, there were two forms of MnS on biochar surface in the aqueous solution, as MnS_(s)_ particles could be dissolved in the water with a reported *K*_s0_ of 10^−13.73^ (Fig. S7†),^18^ and the first form was MnS_(s)_ and the other was MnS_(aq)_. The increased solubility of MnS particles in acid solution may be caused by the proton-promoted dissolution mechanism, and the sequential reactions could be described by the following equations (eqn (1)–(3)). Mn element in MnS_(aq)_ primary existed in the form of MnHS^+^ at weak acid solution (pH = 5.0–6.0). Hence, it favored the reduction of Cr(vi) *via* a combination of homogeneous and heterogeneous at weak acid solution. For the heterogeneous pathway, Cr(vi) could be adsorbed on MnS_(s)_ surface sites, and the surface complexes were formed. Then electron transfer between Cr(vi) and biochar surface or MnS_(s)_ caused the formation of Cr(iii) and Mn(ii) change to Mn(iii).^19^ During this process, S(−ii) played an important role in the Cr(vi) reduction with the formation of S(vi), while Mn element had no change in its valence state. The potential reaction was shown in eqn (4). For the homogeneous pathway, the potential reaction was shown in eqn (5). During the process, hydrolytic MnHS^+^ on MnS_(aq)_ surface would react with Cr(vi) and the products of Mn_2_O_3_ and Cr_2_S_3_ formed. In the reaction, Mn(ii) was involved in the Cr(vi) reduction with the formation of Mn(iii), while S(−ii) had no change. Summary, both biochar support and MnS particles were contributed to the removal of Cr(vi): Cr(vi) could be adsorbed onto biochar support surface through O-containing functional groups, and Cr(vi) would also be reduced by MnS particles *via* homogeneous and heterogeneous pathways at weak acid solution.

1 MnS + H^+^ → MnHS^+^

2 MnHS^+^ + H^+^ → Mn^2+^ + H_2_S_(aq)_

3 H_2_S_(aq)_ ↔ H_2_S_(g)_

4 3MnS_(s)_ + 8HCrO_4_^−^ + 16H^+^ → 3Mn^2+^ + 4Cr_2_O_3_ + 3SO_4_^2+^ + 8H_2_O

5 6MnHS^+^ + 4HCrO_4_^−^ + 4H^+^ → 3Mn_2_O_3_ + 4Cr_2_S_3_ + 7H_2_O

## 4. Conclusions

This paper employed a one-pot method to prepare a new biochar support manganese sulfide adsorbent. This adsorbent could effectively adsorb and reduce Cr(vi) in aqueous solution, and the equilibrium Cr(vi) adsorption amount was about 98.15 mg g^−1^ at pH 5.0–6.0. A part of Cr(vi) could be adsorbed on biochar support surface with the O-containing function groups, while the others would be reduced by MnS nanoparticles *via* homogeneous and heterogeneous pathways at weak acid solution. The synergistic effect between adsorption and reduction/precipitation and the simple preparation process would make this adsorbent a promising material for the purification of Cr(vi)-contaminated wastewater.

## Conflicts of interest

There are no conflicts to declare.

## Supplementary Material

RA-009-C9RA06028F-s001
