# Supplementary material for: Effective removal of Cr(vi) from aqueous solution by biochar supported manganese sulfide
Source: RSC Adv. 2019 Oct 2;9(54):31333–42. doi: 10.1039/c9ra06028f (PMC9072298; doi:10.1039/c9ra06028f)
Supplement: RA-009-C9RA06028F-s001 [file RA-009-C9RA06028F-s001.pdf]

Electronic Supplementary Material (ESI) for RSC Advances.
This journal is © The Royal Society of Chemistry 2019

Data analysis

The initial adsorption rate (h) equation was as follows.

$$h = k_2 q_e^2$$

The normalized standard deviation (NSD) and average relative error (ARE), indicating the validity of kinetic models, are defined as:

$$NSD = 100\sqrt{\frac{1}{N-1}\sum_{i=1}^{N}\left[\frac{(q_{ti}^{exp} - q_{ti}^{cal})}{q_{ti}^{exp}}\right]^2}$$

$$ARE = \frac{100}{N}\sum_{i=1}^{N}\left|\frac{(q_{ti}^{exp} - q_{ti}^{cal})}{q_{ti}^{exp}}\right|$$

where q_{ti}^{exp} and q_{ti}^{cal} (mg/g) are experimental and calculated Cr(VI) adsorbed on MnS@biochar at time *t* and *N* is the number of measurements made. The smaller NSD and ARE values reveal more accurate estimation of $q_t$values. The governing equations of the three models are integrated by applying the boundary conditions $q = 0$ at $t = 0$

The Marquardt's percent standard deviation (MPSD) and the hybrid error function (HYBRID) indicated the validity of adsorption isotherm:

$$MPSD = 100\sqrt{\frac{1}{N-P}\sum_{i=1}^{N}\left(\frac{q_{ei}^{exp} - q_{ei}^{cal}}{q_{ei}^{exp}}\right)^2}$$

$$HYBRID = \frac{100}{N-P}\sum_{i=1}^{N}\left|\frac{\left(q_{ei}^{exp} - q_{ei}^{cal}\right)^2}{q_{ei}^{exp}}\right|$$

where q_{ei}^{exp} is the data from the batch experiment *i* (mg/g), q_{ei}^{cal} is the estimated value from the isotherm for corresponding q_{ei}^{exp} (mg/g), *N* is the number of observations in experimental isotherm, and *P* is the number of parameters in regression model. The smaller MPSD and HYBRID values indicate more accurate estimation of $q_e$values.

Figures

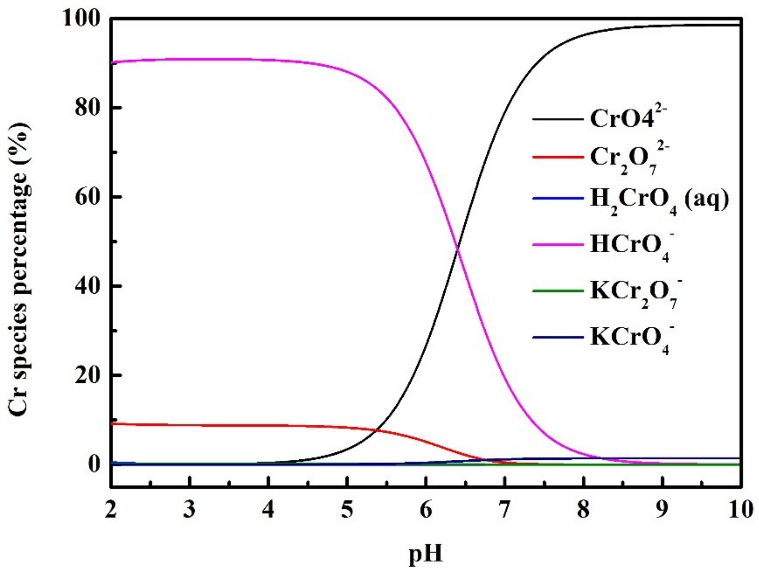

Fig. S1 Cr species percentage as the function of pH (Condition: $C_{Cr(VI)}$=150 mg/L)

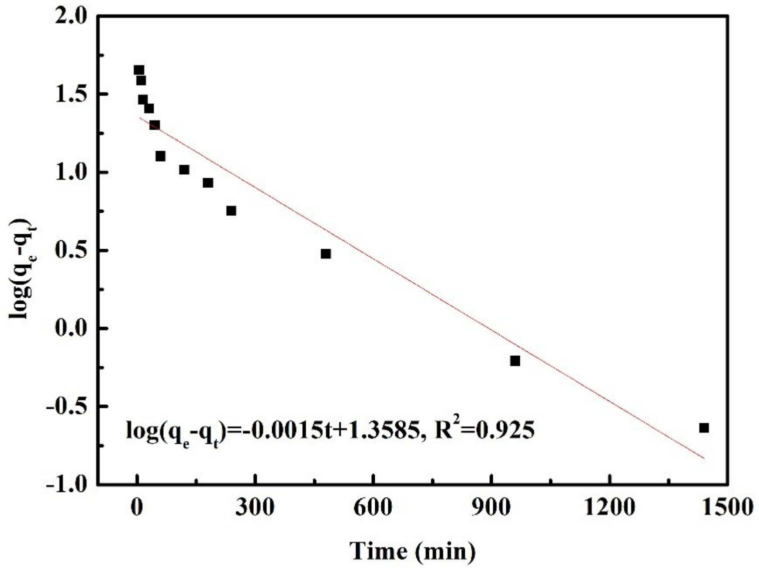

Fig. S2 The pseudo first-order kinetic model fitting line

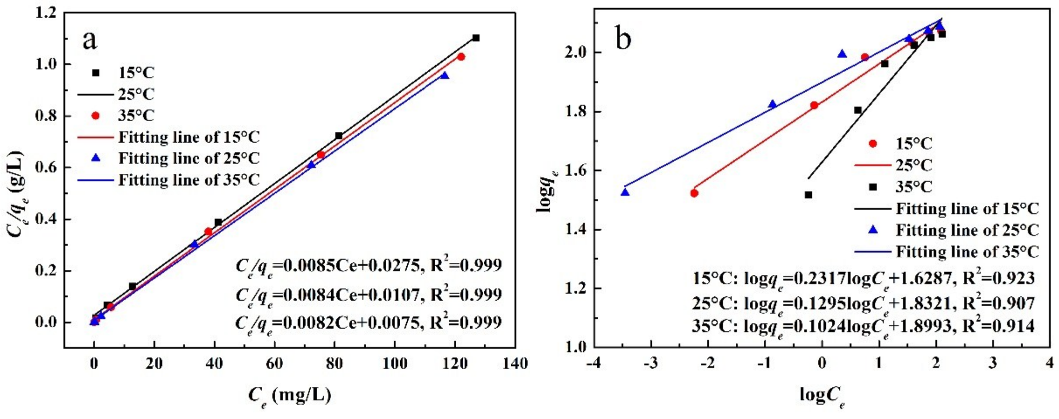

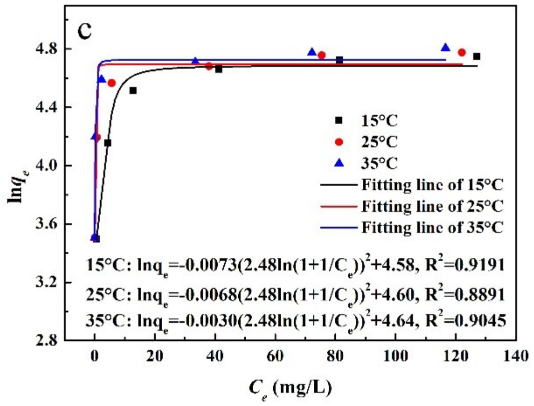

Figure S3 The fitting results of adsorption isotherms models (a: Langmuir model, b: Freundlich model, c: Dubinin-Radushkuvich model)

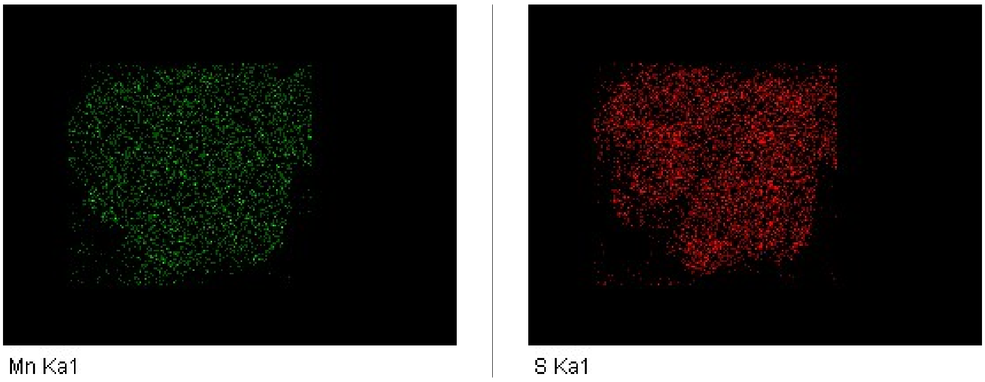

Figure S4 EDS analysis of F-MnS@biochar

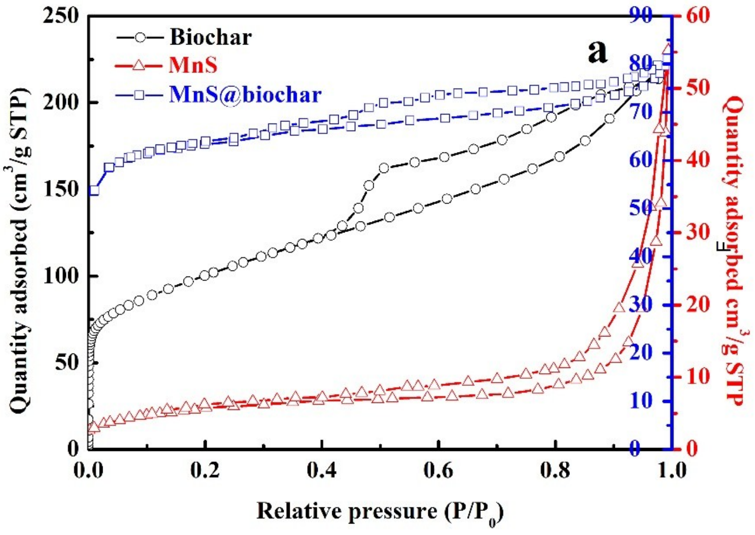

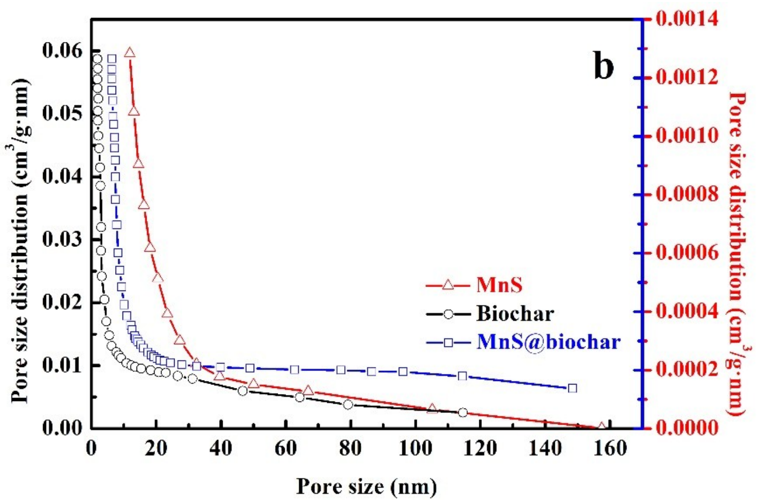

Fig. S5 The specific surface areas (a) and porous structures (b) of MnS, biochar, and F-MnS@biochar

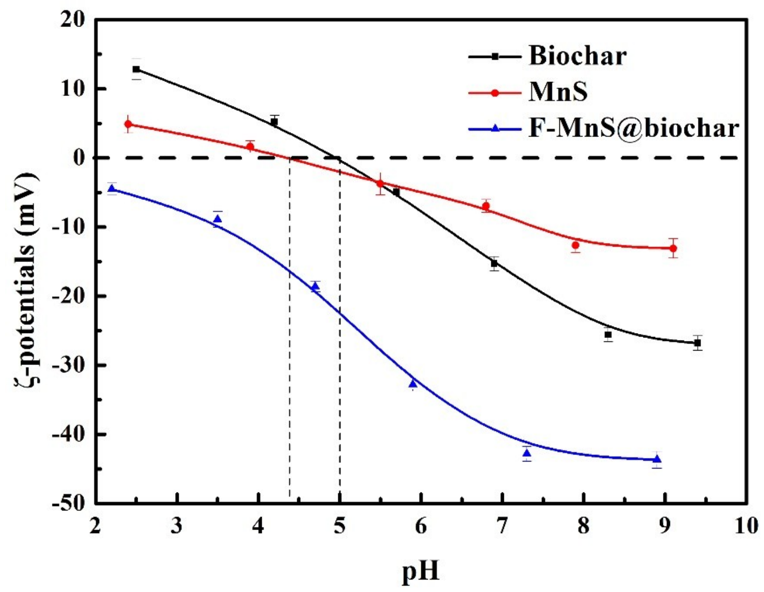

Fig. S6 ζ-potentials of biochar, MnS, and F-MnS@biochar

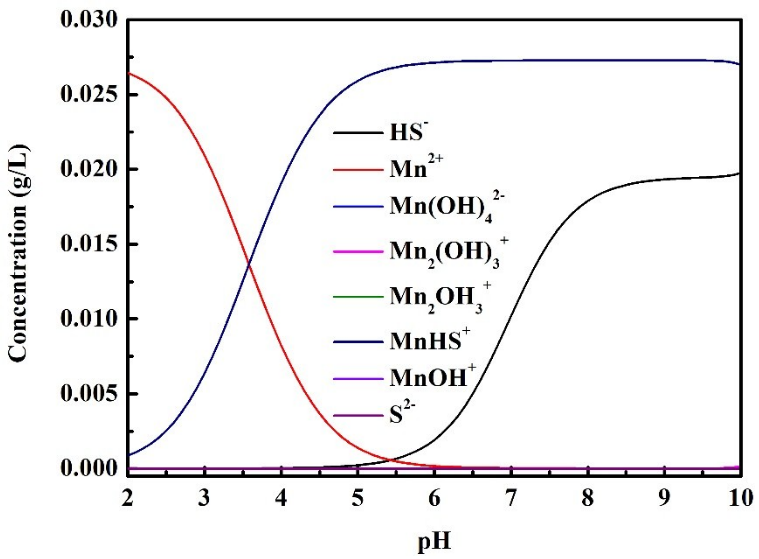

Fig. S7 MnS hydrolysis as the function of pH (Condition: C_{MnS}=1.5 g/L)

Tables

Table S1 The fitting parameters of pseudo first-order model and pseudo second-order model used for simulating Cr(VI) adsorption kinetic data

Model	k_1/k_2	q_e	h_1/h_2	R^2	NSD	ARE
PFO	0.0035	22.83	0.08	0.925	62.1	55.3
PSO	0.0306	98.04	9.02	0.999	11.6	8.7

Table S2 The Cr(VI) removal amounts of different biochar materials

Material	Qe	Ref.
CMC-FeS@biochar	130.5 mg/g	1
ZVI-biochar	17.8 mg/g	2
Fe-biochar	67.44 mg/g	3
β-FeOOH/SYBK	37.04 g kg^{-1}	4
Fe3O4@SiO2-NH2-biochar	27.2 mg/g	5
Fe3O4@biochar	25.27 mg/g	6

Table S3 Regression parameters of adsorption isotherm data of Cr(VI) onto MnS@biochar by Langmuir, Freundlich, Redlich-Peterson, and Dubinin-Radushkevich models

Temperaure	Model	Parameters	MPSD	HYBRID
15° C	Langmuir	q_m=117.65; b=0.3091; R^2=0.999	9.51	4.88
	Freundlich	n=4.3159; k_2=42.53; R^2=0.923	44.75	21.91
	Redlich-Peterson	K_R=93.34; α = 1.27; β= 0.9083; R^2=0.977	4.86	1.48
	Dubinin-Radushkevich	q_m = 97.51 mg/g; K_D = 0.0073; E= 8.28	38.95	17.24
25° C	Langmuir	q_m=119.05; b=0.7850; R^2=0.999	17.35	3.26
	Freundlich	n=7.7220; k_2=67.94; R^2=0.907	38.26	18.73
	Redlich-Peterson	K_R=23178; α = 310; β= 0.8985; R^2=0.982	3.17	0.63
	Dubinin-Radushkevich	q_m = 99.48 mg/g; K_D = 0.0068; E= 8.57	42.33	15.45
35° C	Langmuir	q_m=121.95; b=1.0933; R^2=0.99109	14.02	5.23
	Freundlich	n=9.7656; k_2=79.30; R^2=0.914	40.55	19.28
	Redlich-Peterson	K_R=362792; α = 4280; β= 0.9207; R^2=0.981	5.42	2.19
	Dubinin-Radushkevich	q_m = 103.54 mg/g; K_D = 0.0030; E= 12.91	40.59	16.23

Table S4 XPS results of F-MnS@biochar, and R-MnS@biochar (%)

Samples	O1s				S2p		
	C-O	-OH	C=O	M=O	S(VI)/S(IV)	S(-II)*	S(-II)#
F-MnS@biochar	22.3	60.8	16.9	-	7.6	12.8	79.6
R-MnS@biochar	-	42.7	35.6	21.7	15.3	11.9	72.8
Samples	Mn2p				Cr2p		
	Mn(II)	Mn(III)	Mn(IV)	Me=O	Cr_2O_3	Cr_2S_3	Cr(VI)
F-MnS@biochar	91.6	-	8.4	-	-	-	-
R-MnS@biochar	62.8	29.7	7.5	21.7	24.5	42.8	32.7

References:

1. Lyu H, Tang J, Huang Y, et al. Removal of hexavalent chromium from aqueous solutions by a novel biochar supported nanoscale iron sulfide composite, Chemical Engineering Journal, 2017, 322:516-524.
2. Dong H, Deng J, Xie Y, et al. Stabilization of nanoscale zero-valent iron (nZVI) with modified biochar for Cr(VI) removal from aqueous solution. Journal of Hazardous Materials, 2017, 332:79-86.
3. Duan S, Ma W, Pan Y, et al. Synthesis of magnetic biochar from iron sludge for the enhancement of Cr (VI) removal from solution. Journal of the Taiwan Institute of Chemical Engineers, 2017: S1876107017303516.

4. Yang T, Meng L, Han S, et al. Simultaneous reductive and sorptive removal of Cr(vi) by activated carbon supported β-FeOOH. RSC Advances, 7.

5. Shi S, Yang J, Liang S, et al. Enhanced Cr(VI) removal from acidic solutions using biochar modified by Fe_3O_4@SiO_2-NH_2 particles. Science of the Total Environment, 2018, 628–629: 499-508.

6. Zhang X, Lv L, Qin Y, et al. Removal of aqueous Cr(VI) by a magnetic biochar derived from, Melia azedarach, wood. Bioresource Technology, 2018, 256:1-10.
